# Therapy-induced mRNA, rRNA and tRNA methylation alterations confer tolerance phenotype in tumor cells: mechanism and implications

**DOI:** 10.7150/ijbs.120764

**Published:** 2026-01-01

**Authors:** Anfeng Jiang, Shujie Liu, Zhiyuan Li, Xiongzhou Zhang, Minghao Duan, Bin Li

**Affiliations:** 1 Department of Oncology, Xiangya Hospital, Central South University, Changsha 410008, Hunan, People's Republic of China.; 2 Department of Thoracic Surgery, Xiangya Hospital, Central South University, Changsha 410008, Hunan, People's Republic of China.; 3 Department of Public Health Laboratory Sciences, School of Public Health, Hengyang Medical School, University of South China, Hengyang, 412017, Hunan, People's Republic of China.; 4 National Clinical Research Center for Geriatric Disorders, Changsha 410008, Hunan, People's Republic of China.; 5 Xiangya Lung Cancer Center, Xiangya Hospital, Central South University, Changsha 410008, Hunan, People's Republic of China.

**Keywords:** RNA, methylation, drug tolerant tumor cells, clinical strategies

## Abstract

Drug tolerant persister cells (DTPs) refer to a transient drug-tolerance sub-population of cancer cells characteristics of phenotype plasticity and heterogeneity. This adaptive cell state is a critical transitional phase, standing on the crossroad that cancer cells reacquire drug sensitivity or enter into the permanent drug resistance. Emerging evidences indicate the epitranscriptomic regulations, particularly RNA methylations are the important mechanism underline post-transcriptional regulations of genes expression across all RNA species. RNA is integral to gene expression as messenger RNA (mRNA), transfer RNA (tRNA) and ribosomal RNA (rRNA), which play roles in transmitting information from DNA to the synthesis of functional proteins. Methylation modifications on these RNAs are prevalent and represent a well-recognized non-genetic mechanism, exerting multifaceted regulatory effects on nucleic acid metabolism, such as nucleotide precursor availability, RNA processing dynamics, sub-cellular localization, transcript stability and translational fidelity/ efficiency. This review systematically sorts out the relevant references, demonstrating recent advances on the knowledge of the patterns of methylation modifications on mRNA, tRNA and rRNA, and how these modifications drive the generation and development of DTPs, which hallmarks of epithelial-mesenchymal transition, metabolism shift and immune escape. And then clinical strategies are delineated, leveraging pharmacological modulators of RNA-modifying enzymes alongside non-pharmaceutical lifestyle advice, for the development of therapy strategies preventing DTPs-rooted tumor relapse in this anti-tumor armamentarium with cytotoxic reagents, targeted therapies and immunotherapies.

## 1. Introduction

Early in the 1900s, the initial application of nitro compounds as cytotoxic agents to treat leukemia marked a milestone in systemic cancer therapy. Since then, this therapeutic approach has been evolving continuously, evidenced by recent advancements in oncogene-targeted therapies and T-cells-centered immunotherapies, heralding a new era of precision medicine. However, the current standard treatments confer unprecedented benefits to a minority of cancer patients, while the majority still face incurable disease with enigmatic questions 1.

The most well-recognized challenge in cancer treatment lies in the adaptive drug tolerance of tumor cells. This phenomenon parallels the evolutionary processes observed in organism survival in the strict intracellular and extracellular environments 2. During this adaptive process, non-genetic and genetic alterations are intertwined, with epigenetic regulations emerging as the key determinants of targeted gene expression that underlie cells identities. Among non-genetic variations, chromatin-associated modifications deserve particular scrutiny, and these modifications mainly occur at genomic loci where the primary DNA sequence remains unchanged. These epigenetic variants discriminate between cellular states and harmoniously coordinate with DNA- and RNA-based epigenetic markers, collectively shaping the cellular response to given drugs. Notably, a recent comprehensive review systematically summarizes the pivotal role of extracellular vesicles (EVs) in mediating cancer therapy resistance 3. EVs secreted by drug-resistant tumor cells and cells in the tumor microenvironment confer treatment resistance to neighboring sensitive tumor cells through cargo, including DNAs, RNAs, proteins, and lipids. This EV-mediated intercellular communication enables diverse cancers to acquire therapeutic resistance in chemotherapy, radiotherapy, targeted therapy and immunotherapy.

An increasing number of modifications involving different chemical elements, such as nitrogen (N), oxygen (O) and sulfur (S), have been extensively studied and elucidated**.** In particular, RNA methylation stands out as a significant modification. Dysregulation of RNA methylation processes or mutations in genes encoding methylation regulatory proteins, including RNA methyltransferases “writers”, RNA methylation modification recognition proteins “readers” and RNA demethylases “erasers”, have increasingly been identified as central modifiers that help sustain the tolerance phenotype in tumor cells.

RNA is integral to gene expression as messenger RNA (mRNA), transfer RNA (tRNA) and ribosomal RNA (rRNA), which play distinct roles in transmitting genetic information from DNA into the synthesis of functional proteins. Upon initial drug exposure, molecular rewiring occurs within hours, followed by weeks of epigenetic changes that reinforce and stabilize the adaptive responses to drugs. During this period, a significant decrease in total mRNA abundance and a corresponding global reduction in protein synthesis are observed, reflecting the slow proliferation rate. Notably, a subset of RNA methylations are augmented in translation to sustain the survival of drug tolerant persister cells (DTPs). As a classic transporter, tRNA guides amino acid to rRNA for protein synthesis by matching its anti-codon with the mRNA template. Notably, tRNAs exhibit the highest level of methylation, which provides chemical substrates for cleavage by specific enzymes closely linked to cellular adaptive responses under external stresses. Furthermore, the alterations in the ribonucleoside base modifications within tRNA significantly impact its stability, transport and translation efficiency during the translation process.

In this review, we conduct an in-depth exploration of the methylation processes of mRNA, tRNA and rRNA, and summarize their contributions to the drug tolerance phenotypes of tumor cells to external drugs, including chemotherapy, targeted therapy and immunotherapy. We also discuss small-molecule inhibitors or activators, especially those targeting enzymes such as the "writers", "readers" and "erasers" participating in RNA methylation. Ongoing clinical trials are highlighted, demonstrating the promising designs for treatment modalities that combine with current strategies to combat tumor.

## 2. Overview of RNA methylation

### 2.1 Types of RNA methylation

**Figure 1:**
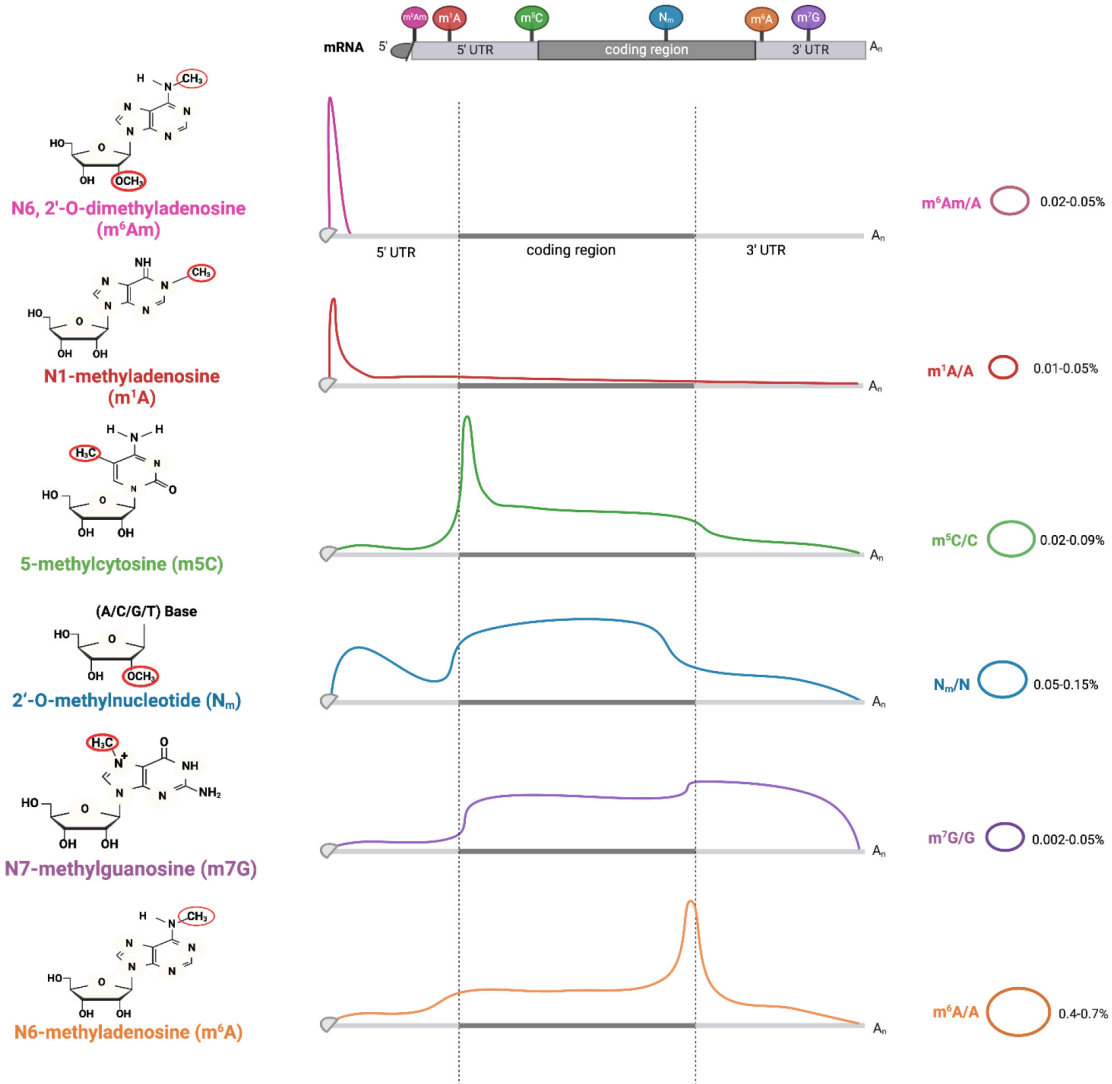
** Methylation Modifications and Their Distribution Patterns in Eukaryotic mRNA.** Starting from nucleotides (adenosine, cytosine, guanine), "writer" enzymes catalyze reactions to produce Nm, m1A, m6A, m5C, m3C, and m7G. Coversely, "eraser" enzymes can remove these modifications. The distribution of m6Am, m5C, Nm, and m6A ranges across various regions of mRNA, from the 5'-UTR, coding region, to 3'-UTR. The relative abundances of these modifications are represented in two ways. The curves show the normalized density distribution of each modification along the mRNA (from 5'-UTR to 3'-UTR), where the height of the curve at a given position reflects how frequently that modification occurs at that location. The percentages (e.g., m6Am/A=0.02-0.05%) indicate the molar ratio of the modified nucleotide to the total amount of the corresponding unmodified nucleotide (e.g., m6Am to total A) across the entire mRNA population analyzed. Image made with BioRender.com.

RNA methylation is a predominant form of epigenetic modification, accounting for over 60% of all RNA modifications 4. The common sites of RNA methylation primarily include N1-methyladenosine (m1A), N3-methylcytidine (m3C), N3-methyladenosine (m3A), 5-methylcytosine (m5C), N6 -methyladenosine (m6A), 7-methylguanosine (m7G) and 2'-O-methylation (Nm) 4 **(Figure 1) (Table 1)**. In the RNA building blocks, the adenosine component can be methylated at distinct nitrogen atoms on its nucleobase (N1, N2 or N6). Moreover, the cytosine, guanine or even the 2' oxygen atom on ribose within these building blocks may also be methylated.

First identified in the 1960s, m1A-denoting adenosine methylation at position 1 is enriched in both the 5'-untranslated region (5'-UTR) of mRNA and various regions within tRNAs. The tRNA methyltransferase 61A (TRMT61A) and methyltransferase-like 1 (METTL1) function writers for m1A modifications. YTH domain-containing protein 1 (YTHDC1) and YTH domain family proteins 1/2/3 (YTHDF1/2/3) act as readers, while alpha-ketoglutarate-dependent dioxygenase alkB homolog 3 (ALKBH3) and fat mass and obesity-associated protein (FTO) function as erasers involved in the m1A demethylation processes.

m3C is notably enriched at position 32 in tRNAThr, tRNASer, and tRNAArg, representing a modification in the tRNA anti-codon loop. In humans, METTL2A, METTL2B and METTL6 catalyze m3C formation, while METTL8 remains ambiguously linked to mitochondrial tRNA modifications or mRNA regulation. In contrast, m3A is a separate modification enriched in rRNA and mRNA, catalyzed by the "writer" METTL5.

m5C is a chemical modification occurring at the fifth carbon atom of cytosine in RNA. The m5C modification sites are often located in a CG-rich environment, mainly in 3'- untranslated regions (3'-UTRs) and coding sequence (CDS) regions of mRNA, with fewer m5C modifications in the 5'-UTR. The NOL1/NOP2/SUN domain (NSUN) family (NSUN1, NSUN7) and tRNA aspartic acid methyltransferase 1(TRDMT1) mainly act as writers, while AlkB Homolog 1 (ALKBH1) and Ten-eleven translocation (TET) families function as erasers, and the Aly/REF export factor (ALYREF) and Y-box binding protein 1(YBX1) serve as major readers 5.

m6A was first identified in mammalian mRNA in the 1970s. The m6A modifications are predominantly localized to the consensus motif RRACH (R=A or G, H=A, C or U), with high enrichment in conserved regions of the 3'-UTRs and near stop codons of transcripts. The readers of m6A encompass diverse molecules such as insulin-like growth factor 2 mRNA-binding proteins 1/2/3 (IGF2BP1/2/3) and YTHDF1/2/3. m7G is an RNA methylation modification occurring at the N7 position of guanine, typically found at the 5' caps and internal sites of mRNA. This modification significantly influences the nuclear export of mRNA.

Nm (where N=A, U, G, C) is a co- or post-transcriptional modification of RNA, where a methyl group (-CH3) is added to the 2' hydroxyl (-OH) of the ribose moiety. 2'-O-methyl ribose can occur on any base and is an abundant and highly conserved modification found at multiple locations in tRNA, rRNA and mRNA. FtsJ RNA 2'-O-methyltransferase 1(FTSJ1), tRNA methyltransferase 44 homolog (TRMT44) and cap methyltransferase 1 (CMTR1) are the human 2'-O-methyltransferases that target specific positions in tRNAs. These enzymes can methylate the residues at C32 and N34 in the anticodon loop of tRNAPhe and tRNATrp, modify residue 44 in tRNASer or alter the N1 (first transcribed nucleotide) of the mRNA caps 6.

### 2.2 Functions of RNA methylations

#### 2.2.1 Methylation modifications on mRNA, tRNA and rRNA

**Figure 2:**
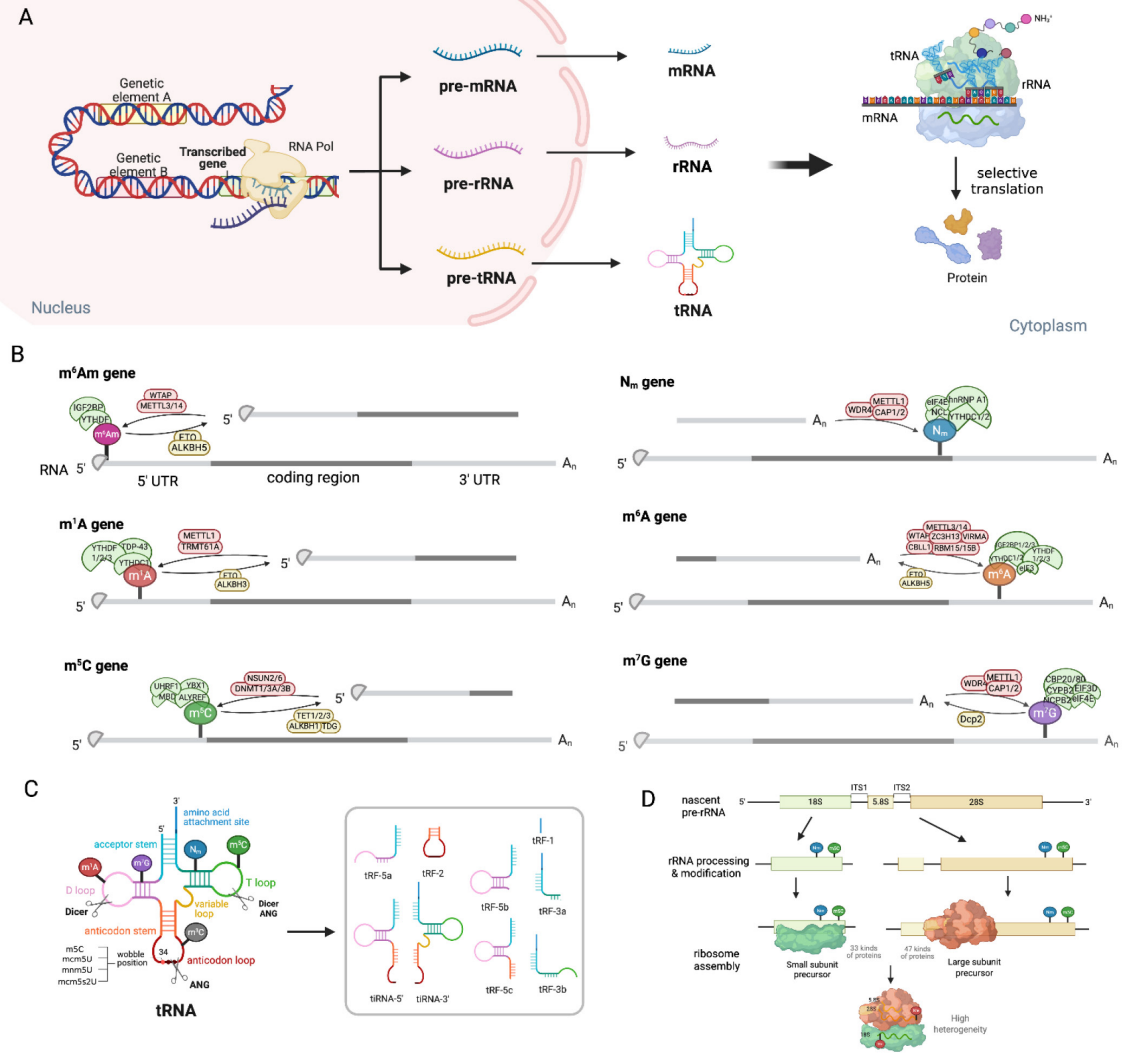
**Modification processes and products in mRNA, tRNA and rRNA. (A)** Genetic elements are transcribed into pre-mRNAs, pre-tRNAs and pre-rRNAs by RNA polymerase. Subsequently, they undergo further process to become mature mRNAs, rRNAs and tRNAs, thereby jointly participating in the protein translation process. **(B)** The representation of different RNA-modifying enzymes, including methyltransferases "Writers" (green), demethylases "Erasers" (yellow), and methylation-reading proteins "Readers"(pink), regulate the processes of m6Am, Nm, m6A, m5C, and m1G.** (C)** Methylation modification in the T loop, D loop and anticodon loop of tRNA clover structure generates various kinds of tiRNAs and tRFs, which play important functions in gene expression. Under drug stress, tiRNAs and tRFs transform into "sharp ammunition", assisting tumor cells in adaptation and survival. **(D)** Nm or m5C modification in ribosomes generate various small subunit (18S rRNA and 33 proteins) and large subunit (5.8S and 28S rRNA and 47 proteins), which assemble into heterogeneous ribosomes. Image made with BioRender.com.

RNA methylation features shared methylation regulators, including erasers (FTO, ALKBH1), readers (YTHDF2) and writers (METTL3/METTL14, TRMT6/61A) that act across mRNA, tRNA and rRNA, forming a unified network integrating global RNA function, as illustrated in **Figure 2**. Among shared erasers, FTO, a member of the AlkB family, can demethylate internal m6A and cap m6Am in mRNA, and N1-methyladenosine (m1A) in tRNA 7-10. Interestingly, FTO-mediated demethylation had a greater effect on the transcript levels of m6A than that of cap m6Am, which is consistent with the fact that m6A is much more abundant. Anomalous m6A/m6Am modification influences the splicing, stability, and conformation/folding of mRNA, as well as its interaction with partners such as proteins or non-coding RNAs (ncRNAs) 11, 12. FTO depletion induces consistent m1A increases in various tRNAs, including tRNAGlu, tRNAHis, tRNAGly, tRNAAsp, tRNALys, tRNAGln, and tRNALeu, fostering translational fidelity, which is consistent with the fact that m1A-methylated tRNAs are preferentially recognized and delivered to translation-active polysomes 7. ALKBH1, another eraser, demethylates m1A/m5C in mRNA to regulate gene expression 13, 14. Notably, as a key oxidase of m5C in RNA, ALKBH1 catalyzes the formation of 5-hydroxymethylcytosine (5-hmC) and 5-formyl cytosine (f5C) from m5C at the wobble site of tRNA, and reprograms the decoding ability of tRNA and then promotes codon-biased translation 15-17. YTHDF2, a reader protein, can bind to m6A/ m1A mRNA to mediate its destabilization 18-20, while binds to m5C rRNA for modulating the maturation of ribosomal RNA 21. Interestingly, the latest research shows that YTHDF2 can function as a dual reader where it stabilizes mRNAs as a m5C reader via overexpressing PABPC1 and enhancing ATP synthesis, as well as destabilizing some mRNAs as an m6A reader 22. TRMT6/61A complex, initially identified as the m1A writer of tRNA 23, installs m1A in stress-responsive mRNAs 24, 25. METTL3/METTL14, the most typical METTL family member, is the core writer for m6A of mRNA 26-28, while METTL2, METTL6, and METTL8 are well-known tRNA m3C methyltransferases. Collectively, these shared regulators form a conserved and interconnected epitranscriptomic network in mRNA metabolism and tRNA/ rRNA function, and therefore controlling translation regulation.

Traditionally recognized as a transfer tool in RNA translation, tRNA has recently been shown to play a critical role in cellular stress responses through its modification-mediated mechanisms. These processes are triggered by stress conditions such as hypoxic sparks, oxidative stress and nutritional deficiencies, which are often induced by anti-tumor drugs. The tRNA harbors numerous rare base modifications 29, and serves as a hotspot for RNA modifications, with methylation being one of the most prevalent chemical modifications. These chemical alterations could be the substrates for cleavage events, giving rise to tRNA-derived stress-induced RNAs (tiRNAs) and tRNA-derived fragments (tRFs), which function potent effectors against cellular stress 30, 31. The m5C modification is particularly enriched in tRNA, occurring at multiple sites including positions 47,48, 49, 50, 71,72, 73, as well as positions 34 and 38 in the anticodon loop 32. Beyond tRNA methylation generating various fragments function as key circuit regulators, these modifications have been demonstrated to play important roles in tRNA folding and other functions. Moreover, the modifications in the anticodon loop are also important for codon-anticodon interplays, therefore impacting the translation process 33. For instance, methylation at positions 37 and 34 (the wobble position) within the anticodon stem-loop is critical for base-pairing interactions that dictate tRNA selection of mRNA codons, ensuring both translation accuracy and efficiency. Modifications outside the anticodon loop impact tRNA folding, thereby influencing its ribosome binding and stability. The main body regions of tRNAs are also frequently modified, which is fundamental to stabilizing the structure and diverse biological functions of tRNA molecules 33. These modifications reduce translation efficacy and detain translational elongation during the targeted protein synthesis, which is usually correlated with the cell cycle and proliferation^34^. Conversely, tRNA reprogramming serves as a cellular adaptive strategy to facilitate the rapid translation of survival-essential proteins 34. Therefore, these methylation modifications on tRNA dramatically influence genes expression through modification-mediated codon-biased translation of stress-response protein-encoding mRNAs, which confer tumor cells adaptive survival (but not proliferation), a state notoriously named drug tolerance.

rRNA undergoes post-transcriptional modification at over 200 nucleotide sites, and its modification profile is tightly coupled with tRNA modifications to govern ribosomal translational capacity in tumor cells. NSUN1-mediated m5C modification at various cytosine residues impacts the rRNA processing and maturation. This modification overlaps with YTHDF2-dependent regulation, as YTHDF2 recognizes m5C loci through the conserved residue Trp432. Deletion of YTHDF2 increases m5C modification sites on 18S and 28S rRNA, thereby affecting rRNA processing, assembly, and ultimately translational fidelity. The modification of Nm protects RNA from hydrolysis and alters RNA flexibility without influencing Watson-Crick base pairing. Despite significant heterogeneity in ribosomal populations, Nm regulates protein synthesis mostly through ribosomal translation control, that has been well-established 35.

#### 2.2.2 RNA methylation-chromatin nexus confer DTPs

The epigenome, extensively observed in cell chromatin, is closely associated with DTPs in targeted therapies or immunotherapies 36, 37. RNAs methylation is considered to exert a significantly influence in gene activation or repression through direct (cis) or indirect (trans) associations with chromatin 38, 39. For example, co-transcriptional m6A of nascent mRNA transcripts recruits the H3K9me2 demethylase KDM3B, which in turn promotes the removal of H3K9me2, leading to global alteration of gene expression 40. Additionally, m6A modification on RNA indirectly influences chromatin modifications by regulating levels of S-adenosylmethionine (SAM), the predominant methyl donor 41. Conversely, high levels of Histone H3 Lysine 36 trimethylation (H3K36me3) at corresponding genomic locations significantly overlap with m6A-modified mRNAs 42, 43. Recent studies suggest that H3K36 trimethyltransferase SETD2 may affect the recruitment of m6A-related enzymes, potentially driving changes in global m6A levels, including those on RNAs 44. METTL14 binds to H3K36me3-modified histones and recruits METTL3 to the sites that are actively transcribed by RNA polymerase II, facilitating co-transcriptional deposition of m6A modification 41, 45. These results indicate the crosstalk between histone and RNA modifications conductive to gene expression^46^.

RNA modification has also been linked to the regulation of DNA methylation. There are observations that a reverse correlation between genome-wide levels of m6A RNA and 5mC DNA. m6A marks deposited by the m6A writer METTL3 can be recognized by the m6A reader FXR1 co-transcriptionally. The presence of FXR1 in turn recruits DNA 5-methylcytosine dioxygenase TET1, leading to global demethylation of DNA, most of which are oncogenes that subsequently become overexpressed and are involved in cancer progression 47. These findings suggest that RNA methylations may not merely be passively modified in response to external treatment stress, but potentially act as active regulator in chromatin histones and/or DNA methylation, which processes are usually reported in DTP evolution 48.

## 3. RNA methylation and DTPs: molecular mechanisms

RNA methylation modifications (m6A, m5C, etc.) dynamically fine-tune transcriptomic diversity, enabling rapid adaptation to therapeutic stress. Research indicates that RNA methylation, specifically m6A, m5C and m3C, can stabilize RNAs within R-loops, thereby inhibiting targeted gene expression 49. Single-cell transcriptomic profiling across tumor models confirms that RNA methylation-driven heterogeneity correlates with Darwinian selection of pre-tolerant clones, where elevated transcriptomic plasticity precedes overt drug resistance 50. Anti-tumor therapies, including recent novel approaches, targeted agents and cytotoxic T cell-centered immunotherapies eliminate malignant cells through diverse mechanisms: disrupting oncogenic signaling or directly damaging tumor cell structure (perforin pores, cytoskeleton degradation, DNA damage, nuclear envelope rupture, reactive oxygen species (ROS)-induced ER stress or mitochondrial stress, et al.) 51.

**Figure 3:**
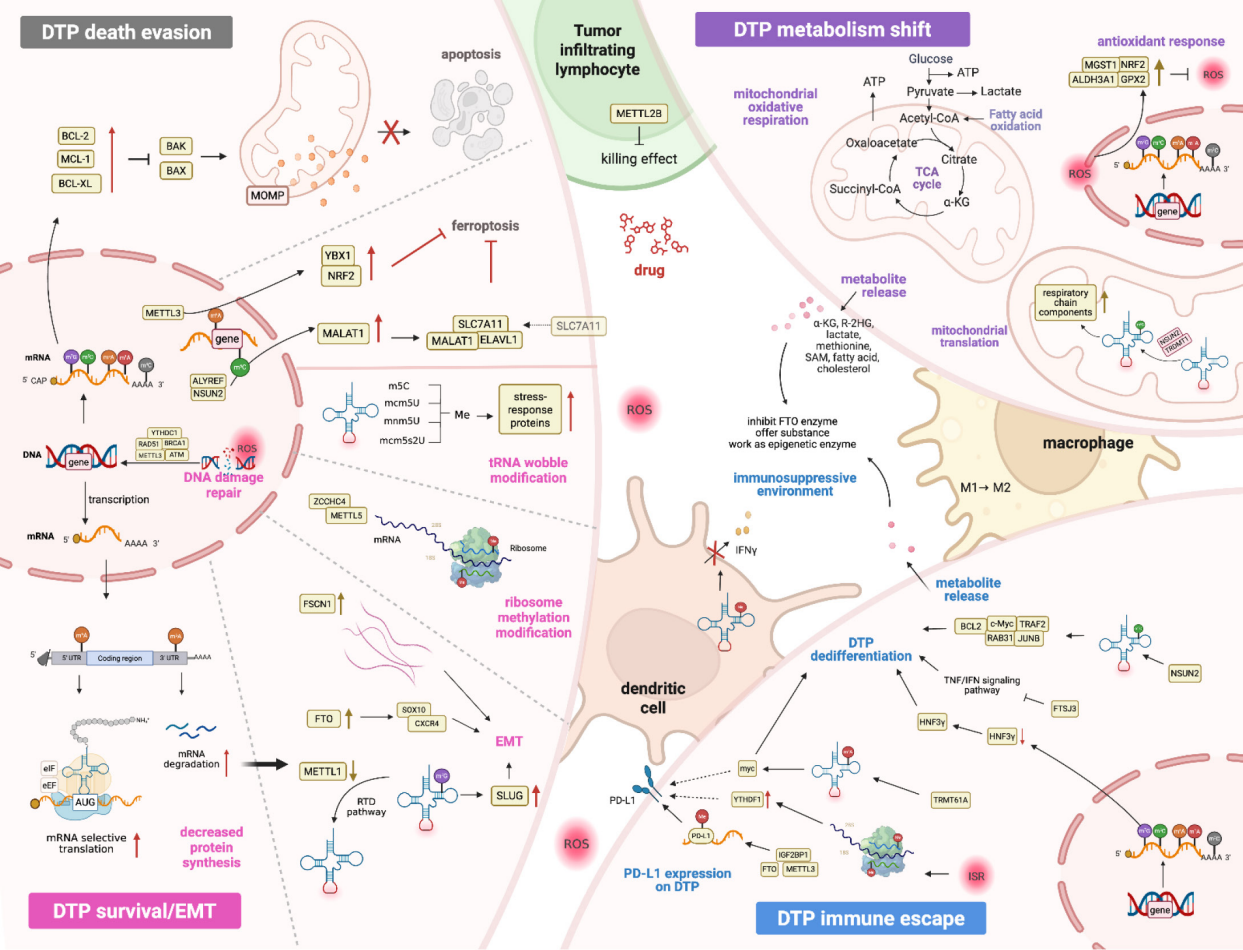
**RNA Methylation facilitates DTPs generation and development. DTP Death Evasion (top left)**: DTPs not only evade apoptosis through the BCL-2 family pathway, but also escape ferroptosis through the involvement of NRF2 and MALAT1, and these processes are regulated by RNA methylation. **Survival/EMT (bottom left)***:* m6A modification on mRNA enhances translation, while this modification on the 3'-UTR promotes mRNA degradation, leading to an overall reduction in protein levels and a selective increase in the translation of crucial mRNAs that is necessary for cells survival. FTO demethylase m6A in CXCR4 and SOX10 for their upregulation, thereby triggering EMT, while decreased METTL1 diminishes m7G-modified tRNAs, mediating EMT via SLUG. Drug stress signal prompt methylation on rRNA and tRNA wobble positions, provoking the stress-response aiding cell survival and EMT. **DTP metabolism shift (top right)***:* Drug stress drives DTPs undergoing significant metabolic changes from glycolysis to oxidative phosphorylation and the corresponding over-activation of antioxidant response mediated by various antioxidant genes (GPX2, NRF2, ALDH3A1, and MGST1), which are driven by epigenetic methylation. tRNA methylation induced by TRDMT1 and NSUN2 regulates mitochondrial translation that underpins metabolic reprogramming in DTPs. **DTP immune escape (bottom right)***:* ISR trigger methylation modification on tRNA or rRNA, causing the enzyme abnormal expression, then enhance tumor PD-L1 expression. Furthermore, these abnormal enzymes also regulation on the mRNA modification of IFN and TNF genes, leading to cells dedifferentiation. Notably, the above processes and the metabolites released by tumor cells also act on the immune cells, including M2 macrophage polarization and dendritic cells inactivation, fostering immunosuppression environments. Image made with BioRender.com.

To survive such insults, tumor cells activate adaptive drug tolerance programs reminiscent of primitive stress-survival strategies observed in unicellular organisms. This survival phenotype is regulated epigenetically through processes akin to "viral mimicry", prioritizes metabolic austerity over proliferation. Under therapeutic stress, DTPs drastically suppress global protein synthesis while upregulating survival-associated protein translation, a coordinated response mediated by amino acid deprivation sensors, unfolded protein response pathways, and inflammatory signaling. These adaptations in cells are convergent to the terminology of “an integrated stress response (ISR)”, which involves modifications of mRNA, tRNA and rRNA and exerts extensive effects on nucleotide supply, splicing, stability, transport, translation efficiency and fidelity 52. Mechanistically, the sublethal release of cytochrome c from the mitochondria of tumor cells triggers activation of heme-regulated inhibitor (HRI), thereby inhibiting the phosphorylation of eukaryotic translation initiation factor 2 alpha (eIF2α), which prevents the recycling of eIF2-GDP to eIF2-GTP, thereby blocking formation of the eIF2-GTP-Met-tRNA ternary complex and suppressing global translation initiation 53-55. This translational arrest is further amplified by stress-induced tRFs, the products of tRNA cleavage, which disrupt eIF4F complex assembly on mRNA cap structures. Concurrently, DTPs sequester untranslated mRNAs in cytoplasmic stress granules (SGs) and stagnate apoptotic proteins, resulting in apoptosis arrest in tumor cells. Therapeutic agents like anthracyclines simultaneously induce oxidative stress, destabilizing the RNA methylome. ROS oxidize RNA modifications (e.g., m5C, m6A) and inhibit methyltransferases (NSUN2, TRDMT1), while activating demethylases (ALKBH1), leading to epitranscriptome-wide hypomethylation 56. This erosion of RNA modifications impairs tRNA decoding fidelity and ribosomal function and exacerbates translational dysregulation. These findings highlight RNA methylation as a central regulator of tumor cell adaptation to external drugs **(Table 2)**, mediating four traits of DTPs, as also shown in **Figure 3**.

### 3.1 RNA methylation mediates death-resistance in DTPs

Cell death encompasses a continuum of molecular states rather than a binary outcome, with DTPs epitomizing the dynamic equilibrium between cell survival and death. Tumor cells near apoptotic thresholds undergo epigenetic reprogramming to evade therapy-induced cell death, demonstrating how sublethal activation of regulated cell death pathways fuels adaptive phenotypic evolution. These epigenetically rewired tolerant cells acquire altered secretory profiles and cell-cell communication networks, disseminating pro-survival signals within the tumor microenvironment to entrench therapy resistance.

Epigenetic reprogramming in DTPs recalibrates translational outputs to prioritize survival over proliferation. A hallmark of this adaptation is the dysregulated equilibrium between pro-apoptotic (e.g., BAX, BAK, BIM) and anti-apoptotic (e.g., BCL2, BCL2L1/BCL-XL, MCL1) family members. Single-cell transcriptomic profiling of epidermal growth factor receptor (EGFR)-mutant lung cancers reveals tumor-specific upregulation of BCL2L1 alongside downregulation of myeloid cell leukemia 1 (MCL1), a stoichiometric imbalance that buffers apoptotic susceptibility 57. Genetic or pharmacological inhibition of BCL2L1/BCL-XL delays acquired resistance, validating this axis as a therapeutic vulnerability in DTPs. Mechanistically, RNA methylation stabilizes anti-apoptotic transcripts (e.g., BCL2) through m6A reader proteins (e.g., YTHDF1), while coordinating with ribosome-tRNA-translation networks to optimize stress-adapted protein synthesis. eIFs and elongation factors (eEFs) synergize with epitranscriptomic modifiers to enforce metabolic quiescence, enabling DTPs to allocate resources to survival under therapeutic duress.

Ferroptosis, an iron-dependent cell death form driven by lipid peroxidation, is subverted in DTPs through RNA methylation-mediated redox adaptation. Sorafenib induces ferroptosis via ROS and Fe²⁺ accumulation, but DTPs counteract ferroptosis by activating the nuclear factor erythroid 2-related factor 2 (NRF2) antioxidant pathway that is mediated by METTL3-dependent m6A modification 58, 59. The RNA methyltransferase NSUN2 installs m5C modifications on NRF2 mRNA, which are recognized and stabilized by YBX1, thereby amplifying NRF2-driven transcription of glutathione synthesis genes, such as solute carrier family 7 member 11 (SLC7A11) and glutamate-cysteine ligase catalytic subunit (GCLC) 60. This NSUN2/YBX1/NRF2 axis establishes a redox buffer that neutralizes tyrosine kinase inhibitor (TKI)-induced lipid peroxidation 61, enabling DTP persistence. The NSUN2/ALYREF complex further reinforces ferroptosis resistance by epitranscriptomic regulation of long ncRNA metastasis-associated lung adenocarcinoma transcript 1 (MALAT1). In sorafenib-resistant tumors, m5C methylation stabilizes MALAT1, which scaffolds a ternary complex with ELAVL1 and SLC7A11 mRNA to enhance cystine import and glutathione synthesis. This MALAT1/ELAVL1/SLC7A11 feedforward loop sustains redox homeostasis, while NSUN2-mediated tRNA m5C modifications ensure efficient translation of ferroptosis defense proteins 62. Pharmacologic disruption of NSUN2-ALYREF-MALAT1 signaling restores sorafenib sensitivity, positioning this pathway as an actionable target for overcoming TKI-tolerant tumor cells 62.

### 3.2 RNA methylation mediates DTP survival/EMT

RNA epitranscriptomic reprogramming governs the survival-death equilibrium in DTPs through spatial regulation of mRNA translation and stability. Stress-primed DTP subpopulations overexpress the activating transcription factor 3 (ATF3) to prioritize selective translation of survival transcripts (e.g., HIF1α, ATF4) via 5ʹ-UTR m6A-eIF4A axis activation, whereas 3ʹ-UTR m6A-YTHDF2 interactions degrade proliferation drivers (e.g., MYC) 63. This methylation-driven translational plasticity enables metabolic adaptation and oscillatory gene expression, characteristics of BRAF/MEK inhibitor-persistent melanomas 63. Concurrently, FTO-mediated m6A erasure upregulates CXCR4 and sex-determining region y box 10 (SOX10) to drive epithelial-mesenchymal transition (EMT), conferring the resistance to programmed cell death protein 1 (PD-1) 64. Therapeutic inhibition of FTO restores m6A levels and reverses immunotherapy tolerance 65, validating epitranscriptomic editing as a strategy to overcome adaptive persistence. Single-cell transcriptomic profiling reveals four distinct epigenetic states in melanoma cell lines and patient tumors, independent of genomic alterations. These transcriptional programs arise from coordinated RNA methylation dynamics that enable adaptive survival under therapeutic pressure. Highly translated mRNAs exhibit 5'-UTR-enriched m6A modifications that promote ribosomal engagement, while 3'-UTR m6A hypomethylation stabilizes stress-response transcripts by evading YTHDF2-mediated decay. Such spatially regulated epitranscriptomic rewiring upregulates pro-survival genes (e.g., ATF4, HIF1A) while silencing proliferation drivers (e.g., MYC, CCND1), a hallmark of BRAF/MEK inhibitor-persistent melanoma cells. This methylation-driven translational plasticity allows metabolic adaptation and drug tolerance through sustained oscillatory gene expression patterns.

Therapeutic stress activates DNA damage response (DDR) pathways that converge on RNA methylation to sustain genomic integrity. Activation of the ataxia-telangiectasia mutated (ATM)-METTL3 axis coordinates DNA repair with translational control under therapeutic stress. Drug-induced R-loops recruit ATM-phosphorylated METTL3, which deposits m6A marks on nascent RNA to recruit YTHDC1 66, RAD51 recombinase (RAD51) and breast cancer gene 1 (BRCA1). This METTL3-YTHDC1-RAD51/BRCA1 axis stabilizes RNA:DNA hybrids while suppressing replication stress, enabling DTPs to maintain genomic integrity under genotoxic pressure. In parallel, miR-143-3p/miR-145-5p target m6A-modified fascin actin-bundling protein 1 (FSCN1) to disrupt focal adhesion-ECM mechanotransduction 67, further inducing therapy tolerance. These findings position RNA methylation as a molecular bridge linking DNA damage response to cytoskeletal remodeling in support of the metastatic potential of the DTPs.

Epigenetic-epitranscriptomic interplay further promotes drug tolerance through EMT and chromatin remodeling. Elevated histone H3K27me3 at the promoters of m6A-modified mRNAs synergizes with METTL3 overexpression to drive EMT via Snail/Slug upregulation. The tRNA/rRNA epitranscriptome fine-tunes translation initiation rates through codon-biased decoding, while stress-induced tRNA fragments (e.g., Leu-CAG) sequester eIF4A to prioritize survival transcripts. Notably, the key regulatory nodes, particularly those governing the coordination of nuclear RNA processing with cytoplasmic translation during adaptive persistence, remain unclear. Dynamic tRNA wobble modifications reprogram codon-biased translation to enforce EMT-driven resistance. NSUN2 loss induces angiogenin-mediated cleavage of m5C-deficient tRNA-Val-AAC, generating tsRNAs that suppress global translation while activating ATF4-driven stress adaptation. Conversely, METTL1-mediated m7G tRNA modifications optimize SLUG/SNAIL translation to sustain metastasis 68, whereas METTL1 depletion triggers CLP1-dependent tRNA decay and ZAKα-eIF2α ribotoxic stress. Ribosomal heterogeneity, shaped by zinc finger CCHC-type containing 4 (ZCCHC4)/METTL5-catalyzed m6A in 28S/18S rRNA, enables preferential translation of redox regulators (glutathione peroxidase 4, GPX4) and DNA repair factors, conferring cross-resistance to both chemotherapy and immunotherapy. Ribosomal heterogeneity, shaped by conserved m6A modifications in 28S (e.g., A4220) and 18S (e.g., A1832) rRNA, catalyzed by via ZCCHC4 and METTL5, diversifies translational output in therapy-tolerant cells. Such specialized ribosomes preferentially translate transcripts encoding redox regulators and DNA repair factors, enhancing resilience against treatment-induced oxidative and genomic stress. Hybrid epithelial-mesenchymal states arising from this epitranscriptomic-ribosomal crosstalk exhibit cross-resistance to chemotherapy, TKIs and immunotherapy. Pharmacologic inhibition of CX-5461-induced rRNA synthesis reverses EMT and restores lineage-specific differentiation. Targeting tRNA/rRNA modifiers, such as inhibiting METTL1 to block metastasis or antagonizing ZCCHC4 to disrupt stress-adapted translation, may reverse the plasticity-driven tolerance phenotype in advanced malignancies.

The p53 signaling integrates epitranscriptomic and chromatin remodeling inputs to modulate EMT plasticity 69. Truncated isoforms (Δ40p53, Δ133p53) recruit polycomb repressive complexes to silence E-cadherin and upregulate ZEB1/TWIST, driving chemoresistance in colorectal models 70. Hyperactivated ERK1/2 phosphorylates ZEB1, stabilizing its interaction with Δ40p53 and amplifying miR-200 repression and vimentin expression. This ERK-ZEB1-p53 axis enhances transforming growth factor beta (TGF-β)/WNT signaling, establishing epigenetic barriers characterized by H3K27me3 enrichment at m6A-modified promoters 71. Therefore, multidimensional modulation of cell-state transitions, particularly targeting splice variants with antisense oligonucleotides or disrupting energy barriers between phenotypic “valleys” to dismantle feedforward loops, may lock tumors in therapy-sensitive states.

### 3.3 RNA methylation avails DTP metabolism shift

Mitochondria serve as a metabolic hub that coordinates energy production and biosynthetic pathways, and their functional plasticity profoundly impacts the therapeutic survival ability of DTPs. DTPs undergo a metabolic shift from Warburg effect-driven glycolysis to enhanced oxidative phosphorylation to maintain low energy demand 72. This transition may be driven by epigenetic factors (such as DNA/RNA methylation) and transcriptional reprogramming 72. Treatment-induced ROS upregulate antioxidant genes (GPX2, ALDH3A1 and MGST1), further reshaping RNA modification landscapes, particularly m6A and m5C marks, to sustain redox homeostasis 73, 74. For instance, changes in the RNA epitranscriptome enhance the expression of NRF2, enabling tumor cells to counteract ROS-mediated metabolic stress and survive. In lung cancer DTPs, the ferroptosis inducer RSL3 significantly kills DTPs by increasing the accumulation of lipid ROS and ferrous ions, a process reversible by iron chelators 75. Interestingly, polyamine metabolism and one-carbon metabolism (the folate cycle, methionine cycle, and the transsulfuration pathway) provide the methyl donor SAM for RNA/DNA methylation, and alterations in these pathways may affect DTP persistence through methylation modifications 76, 77.

RNA methylation, especially m6A and m5C, influences gene expression by regulating RNA metabolism, including stability, splicing, and translation, thereby orchestrating adaptive responses in DTPs. RNA methylation regulates the expression of DTP-related genes by modifying lncRNAs or mRNAs 78. For example, m6A modification can activate the HIF-1α stress response pathway, facilitating cancer cells to enter a dormant state under drug pressure 79. Methylation of mRNAs related to mitochondrial metabolism, such as genes of the electron transport chain (ETC) complex, may promote DTPs to shift towards alternative energy pathways 80. Notably, the crosstalk between tRNA modifications and mitochondrial translation underpins metabolic reprogramming in DTPs. Under ROS stress, m5C at the wobble position of tRNA-Leu-CAA optimizes leucine codon decoding, ensuring accurate translation of mitochondrial respiratory chain components. Concurrently, the TRDMT1-mediated m5C methylation at tRNA-Asp-C38 governs aspartate-rich protein synthesis 81, with its interaction with glycyl-tRNA synthetases during oxidative damage fine-tuning translation fidelity. Pharmacological inhibition of tRNA m5C modification by azacytidine, a process dependent on TRDMT1 methyltransferase activity, disrupts this adaptive mechanism 82, 83. Similarly, NSUN2-driven m5C marks on tRNAs regulate mitochondrial RNA translation, directly impacting tricarboxylic acid cycle flux and electron transport chain efficiency 84. These modifications collectively reprogram mitochondrial metabolism to favor drug tolerance, highlighting tRNA epitranscriptomics as a critical determinant of tumor metabolic plasticity. Taken together, RNA methylation drives the metabolic reprogramming of DTPs by regulating the stability and translation efficiency of metabolism-related RNAs. Conversely, the metabolites, such as the methyl donors, can impact RNA methylation states, forming feedforward loops that provide a theoretical basis for developing combination therapies.

### 3.4 RNA methylation promote immune escape

RNA methylation serves as a critical modulator of immune tolerance in both physiological and pathological contexts, encompassing early embryogenesis to immunodeficiency disorders like sepsis 85, 86. In cancer biology, multiple RNA modifications, including m1A, m5C, m6A and m7G, orchestrate the functions of immune cells, as well as their interactions with the surroudings. RNA methylation reprograms the tumor-immune interface through dual regulation of tumor cells and infiltrating immune cells. In innate immunity, multiple genes encoding type I interferon-related genes are subject to m6A-mediated stability regulation as well including the IFNB1 transcript. Depletion of the m5C writer NSUN2 leads to increased stability of IRF3, resulting in amplification of the type I interferon response. In terms of adaptive immunity, thymocyte differentiation, activation-induced death of peripheral T cells and regulatory T cell function are dependent on WTAP and m6A methyltransferase functions. In dendritic cells, transcripts encoding lysosomal proteases are subject to m6A modifications 87, 88**.** The demethylases (e.g., FTO) suppress cytokine pathways and establish immunosuppressive niches 64, 65, 89, 90. Pharmacological inhibition of METTL3 or FTO restores T-cell infiltration and interferon gamma (IFNγ) sensitivity that is observed in preclinical models, highlighting the therapeutic avenues of epitranscriptome targets. Concurrently, non-m6A modifications such as TRMT61A-mediated tRNA m1A58 and NSUN2-driven tRNA m5C34, specifically regulate the translation of immunoproteasomes and antigen-presenting machinery, further potentiating immune tolerance 91, 92. Collectively, RNA methylation operates as a master regulatory node governing the dynamic equilibrium between tumor immunosurveillance and escape.

Clinically, the methylation risk signature (MRS) integrating these differential modifications robustly predicts patient survival, immune infiltration patterns, and chemotherapeutic sensitivity in patients with gastric cancer (GC) 93. Similarly, epitranscriptomic regulation extends to glioma, colon cancer, hepatocellular carcinoma (HCC), and clear cell renal cell carcinoma, suggesting conserved RNA methylation-driven pathways in immune evasion. Tumors stratified by m6A modification levels exhibit distinct immune phenotypes and therapeutic vulnerabilities. Low-m6A-score subtypes are characterized by enhanced cytotoxic T-cell infiltration, early tumor-node-metastasis (TNM) stages, and favorable prognosis, reflecting an immunologically "hot" microenvironment. In contrast, high-m6A-score tumors exhibit stromal activation, immunosuppressive cytokine profiles, and aggressive progression 94. In cervical cancer and melanoma, m6A-based classifiers predict the responsiveness to PD-1/PD-L1 blockade, with low-m6A level showing superior clinical benefit 95, 96. These findings establish RNA methylation as a universal rheostat balancing immune adaptability during both development and malignancy, and underscore its potential as a pan-cancer biomarker for guiding immunotherapy strategies.

#### 3.4.1 RNA methylation upregulates PD-L1 expression on tumor cells

PD-L1, an immune checkpoint protein overexpressed in human malignancies, facilitates tumor immune evasion by binding PD-1 on T cells to suppress antitumor activity. Post-transcriptional regulation plays a pivotal role in controlling PD-L1 mRNA stability and protein expression. The m6A modifications within the 3'-UTR or CDS of PD-L1 mRNA are dynamically regulated by the methyltransferase METTL3 and the reader protein IGF2BP, thereby enhancing PD-L1 mRNA stability and translation efficiency and amplifying immunosuppressive signaling 97, 98. Furthermore, the ISR during DTP states induces ribosomal methylation, accelerating PD-L1 protein synthesis and membrane localization 99.

RNA methylation exhibits broad clinical relevance in PD-L1-mediated immunotherapy resistance. Elevated m6A levels correlate with PD-L1 upregulation in head and neck squamous cell carcinoma (HNSCC), breast cancer, and gastric cancer. The m6A demethylase FTO enhances melanoma-intrinsic PD-1, CXC chemokine receptor type 4 (CXCR4) and SOX10 expression by erasing m6A marks, while simultaneously suppressing IFNγ response and anti-PD-1 efficacy 64. Clinically, high expression of the m6A reader YTHDF1 predicts poor immunotherapy outcomes in cancer patients due to PD-L1 overexpression dependency 56. These findings position RNA methylation as a central regulator of adaptive immune resistance across malignancies.

Metabolic-epigenetic crosstalk further modulates PD-L1-driven immune evasion. Methionine metabolism supplies methyl donors for RNA methylation, linking nutrient availability to PD-L1 expression 100. Dietary methionine restriction or pharmacological FTO inhibition enhances IFNγ sensitivity and CD8+ T-cell infiltration, overcoming anti-PD-1 resistance 100. Concurrently, TRMT61A-mediated tRNA m1A58 methylation upregulates MYC and PD-L1 synthesis, conferring resistance to oncolytic virus therapy 101. Therapeutic strategies targeting RNA methyltransferases (e.g., METTL3 inhibitors) or tRNA-modifying enzymes (e.g., TRMT61A antagonists) disrupt PD-L1 immunosuppressive networks, indicating metabolic interventions combined with checkpoint blockade may restore antitumor immunity. By integrating epitranscriptomic and metabolic modulation, these approaches provide a roadmap for precision immunotherapy in PD-L1-high tumors.

#### 3.4.2 RNA methylation assists tumor cell dedifferentiation

Tumor cells dynamically reprogram their epigenetic landscapes and phenotypic states during therapeutic interventions, enabling the transitions between differentiated, de-differentiated, and trans-differentiated states. RNA methylation critically regulates these phenotype shifts by activating interferon (IFN) and tumor necrosis factor (TNF) signaling pathways. These pathways promote dedifferentiation and confer immunotherapy tolerance, generating cells with transcriptional signatures resembling "basal-like" characteristics, despite the absence of definitive lineage markers. Recent advances highlight RNA methylation as a dual regulator of transcriptional and translational reprogramming, particularly through epigenetic control of Myc proteins 102, 103. These mechanisms provide a unified framework for understanding tumor plasticity and therapeutic resistance across different cancer types 104.

Therapeutic pressure induces dynamic RNA methylation remodeling, orchestrating tumor cell state transitions through coordinated transcriptional and translational reprogramming. Stress-induced dedifferentiation is characterized by enriched m6A modifications within the 5'-UTRs of highly translated mRNAs 105, 106, a molecular signature strongly associated with TKI tolerance. Integrated genomic analyses further reveal that drug-tolerant tumor cells exhibit downregulation of IFN-related genes, establishing a mechanistic link between suppressed IFN signaling and therapeutic evasion. For example, in HCC, the RNA methyltransferase FTSJ3 promotes immune escape by suppressing double-stranded RNA-triggered IFNβ signaling via its 2'-O-methyltransferase activity 107. Simultaneously, m6A-dependent regulation of HNF3γ drives HCC dedifferentiation and confers sorafenib insensitivity, illustrating how epitranscriptomic modifications enable adaptive survival under therapeutic stress 108. RNA methylation operates through dual regulatory axes, including transcriptional control via IFN/TNF pathway modulation and Myc protein stabilization, as well as translational fine-tuning via selective mRNA stabilization and tRNA-mediated metabolic adaptation 109-111. Notably, in BRAF/MEK inhibitor-treated persister cells, reversible epitranscriptomic rewiring mediated by eIF4A-dependent translation control highlights the dynamic adaptability of these pathways 112. In anaplastic thyroid carcinoma, upregulation of the tRNA m5C methyltransferase NSUN2 correlates with tumor dedifferentiation and aggressiveness 92. NSUN2 catalyzes m5C modifications that stabilize tRNA structure, enhance leucine transport, and maintain proteostasis. These modifications sustain oncogenic drivers such as c-Myc, BCL2, and TRAF2. This finding links tRNA methylation to metabolic-epigenetic crosstalk, as NSUN2 integrates amino acid availability with oncogenic transcription, exemplifying how RNA methylation bridges cellular metabolism and malignant progression.

The convergence of RNA methylation mechanisms on tumor cell state underscores their therapeutic targeting potential. Targeting RNA methyltransferases represents a promising strategy to counteract dedifferentiation-driven therapeutic resistance. FTSJ3 and NSUN2 exemplify context-dependent methyltransferases, and the inhibition of key nodes such as FTSJ3-IFN crosstalk or NSUN2-mediated tRNA modifications may reverse cell dedifferentiation and resensitize tumors to immunotherapy or targeted agents 92, 107. Single-cell spatial mapping of methylation dynamics and metabolic fluxes will be critical for decoupling these context-dependent effects. Collectively, disrupting RNA methylation networks may restore differentiation trajectories, offering a paradigm shift in overcoming adaptive resistance across cancer types.

#### 3.4.3 RNA methylation facilitates immunosuppressive environment

The immunosuppressive tumor microenvironment emerges through synergistic interactions between RNA methylation-mediated immune dysfunction and tumor cell adaptation. Therapy-induced reprogramming of the RNA methylome, particularly through m6A modifications, drives immunosuppression across immune cell populations. Dendritic cells and macrophages adopt tolerogenic phenotypes via m6A-dependent stabilization of hypoxia-response transcripts such as HIF-1α and selective translation of glycolytic enzymes that fuel anti-inflammatory cytokine production 113. Concurrently, cytotoxic lymphocytes (CTLs) undergo functional exhaustion as m6A modifications promote decay of T cell activation markers including CD28 and inducible costimulator (ICOS) while stabilizing immune checkpoint mRNAs like PD-1 and CTLA-4 114. This bidirectional epitranscriptomic regulation establishes a self-perpetuating cycle of immune evasion. METTL2B overexpression in tumor-infiltrating leukocytes simultaneously suppresses antitumor immunity and enhances platinum sensitivity through cell-type-specific RNA stability regulation. In lymphocytes, METTL2B promotes YTHDF2-mediated decay of cytotoxic effector transcripts such as perforin and granzyme B 115, whereas in ovarian tumor cells, METTL2B stabilizes DNA repair factors including BRCA1 and FANCD2 via IGF2BP3-dependent mRNA protection 116. Single-cell transcriptomic profiling reveals spatial co-distribution of METTL2B-overexpressing tumor cells with exhausted CD8+ T cell clusters, demonstrating microenvironmental co-evolution driven by epitranscriptomic crosstalk. These findings necessitate the development of therapeutic strategies that temporally decouple the opposing effects of METTL2B. This means suppressing METTL2B activity in immune cells to restore cytotoxicity, while transiently enhancing METTL2B function in tumor cells to exacerbate platinum-induced DNA damage.

Therapeutic interventions further reshape the epitranscriptomic landscape through genomic instability and metabolic rewiring. Treatment-induced copy number variations dysregulate m7G methylation machinery, with pan-cancer conservation of m7G regulator co-expression patterns reflecting evolutionary selection for RNA modification systems that buffer therapeutic stress 117. Concurrently, tRNA Gm18 2'-O-methylation generates immunosuppressive tRNA fragments that impair interferon responses, synergizing with m6A-mediated immune checkpoint regulation to establish immunotherapy tolerance 118, 119. Metabolic adaptations in drug-tolerant persisters interface with RNA methylation through three interconnected pathways. Firstly, methionine adenosyltransferase-derived SAM sustains METTL3-METTL14 activity via autoregulatory SAM binding, preferentially directing methyl groups toward RNA rather than DNA or histone modifications under mTORC1 regulation 120. Secondly, hypoxia-induced α-ketoglutarate accumulation activates FTO-mediated m6A demethylation while stabilizing DNMT3A/B-dependent m5C modifications, creating bifurcated RNA methylation outcomes 121, 122. Thirdly, the oncometabolite R-2-hydroxyglutarate competitively inhibits α-ketoglutarate-dependent dioxygenases, trapping FTO in an inactive state that preserves immunosuppressive m6A signatures 123, 124. These metabolite-epitranscriptome interactions remodel immunogenic RNA landscapes, deplete nutrients essential for tumor-infiltrating lymphocytes, and stabilize PD-L1 transcripts, collectively reinforcing an immune-evasive niche.

## 4. Clinical translation feasibility

DTPs remain a long-standing and yet unresolved challenge in oncology, even in the new era of oncogene-targeted therapies and immunotherapy. Tumor regression often reaches a plateau, indicating the presence of minimal residual disease (MRD). Evidence supports that sublethal activations of apoptotic pathways are detected in MRD, where CTLs induce repairable damages in tumor cells 51, 55, 125, 126. Therefore, there is an unmet need to explore RNA methylation as a therapeutic target, either alone or in combination with TKIs or immunotherapy, to eliminate residual tumor cells.

### 4.1 Methodology advances

Over the past decade, methodological advancements have facilitated transcriptome-wide profiling of RNA modifications. Currently, there are four major sequencing technologies, including antibody-based technology, reverse transcription signature-dependent technology, enzyme-dependent sequencing techniques and chemical-assisted techniques. First, antibody-based technologies are widely employed for modifications such as m6A, m5C and m7G. Second, reverse transcription signature-dependent techniques rely on mismatches, insertions or deletions (indels) and/or truncations generated at the modification sites. Third, enzyme-dependent sequencing techniques utilize the recognition and catalytic capability of the enzymes (such as demethylases, endonucleases and exonucleases) to discriminate modification sites from regular RNA bases and chemical-assisted techniques. Fourth, chemical-assisted techniques combine chemical treatments with next-generation sequencing (NGS) to enable absolute quantification at single-base resolution. Additionally, spatial omics, single-cell and single-molecule methods address this possibility from both spatial and temporal dimensions 52** (Table 3)**.

### 4.2 RNA methylation-targeting agent exploration

RNA methylation-targeting agents disrupt the survival of tumor cells by modulating three core components of the epitranscriptomic machinery that include demethylases (FTO), methyltransferases (METTL3, PRMT5) and oligonucleotide-based regulators of methylation enzymes.

These methylation-targeted drugs include inhibitors, agonists, degraders and multitarget agents. Several FTO inhibitors have been proven to harbor strong potential for clinical application, including R-2-hydroxyglutarate (R-2HG), Bisantrene (CS1), Brequinar (CS2) and FB23/23-2 90, 127. R-2HG, a metabolite of IDH1/2 mutations, selectively binds the FTO active site to inhibit demethylase activity 124. In FTO high cancer cells, including leukemia and glioma, R-2HG inhibited cancer cell proliferation/survival and promoted cell cycle arrest and apoptosis, while reversing drug resistance by targeting the FTO/m6A/MYC/CEBPA signaling pathway 124. Specifically, R-2HG inhibited FTO activity, thereby increasing global m6A RNA modification and reducing the stability of MYC/CEBPA transcripts in R-2HG-sensitive leukemia cells, showing antitumor activity. CS1 and CS2 could occupy the catalytic active site of FTO and prevent the m6A modified RNA from entering the pocket, thereby inhibiting the m6A demethylase activity of FTO 90. Both of them inhibit FTO by binding to the key amino acid residues of FTO. CS1 binds His231 and Glu234, while CS2 binds Lys216, Ser229 and His231. Since these sites are simultaneously essential for FTO to bind m6A modified RNA, this ensures inhibitory specificity. Furthermore, the FTO inhibitor FB23/FB23-2 can effectively inhibit the proliferation and migration of cancer cells by suppressing FTO 128-130. Mechanistically, FB23-2 inhibits the expression of Erb-b2 receptor tyrosine kinase 3 (ERBB3) and human tubulin β class Iva (TUBB4A) by increasing the level of m6A. The reduction in ERBB3 expression leads to the inhibition of the Akt-mTOR signaling pathway, thereby impairing the proliferation and survival of liver cancer cells.

Some METTL3 inhibitor have also been proven to exhibit the good anti-tumor activity. For example, RM3 inhibits METTL3 activity while also facilitating its proteasomal degradation, and RSM3 increases METTL3 degradation 131. RSM3 can induce the upregulation of genes related to programmed cell death, while inhibiting oncogenic signals. In NAFLD-HCC, targeting METTL3 with single-stranded RNA, nanoparticle siRNA, or drug inhibitors (STM2457) in combination with anti-PD-1 agents can synergistically activate cytotoxic CD8+ T cells and mediate tumor regression 132. Recently, STC-15, the first METTL3 inhibitor, has been approved for use in a 1b/2 phase clinical study 133, 134. The ongoing STC-15 clinical trial includes NCT06975293 (a Phase I/II trial of STC-15 combined with teprotumumab (anti-PD-1) for metastatic NSCLC/melanoma/HNSCC). In addition, there is a clinical trial (NCT06762925) targeting METTL3 peptide inhibitors for urinary tract tumors, which aims to reshape the tumor microenvironment and enhance the anti-tumor immune response **(Table 4)**.

PRMT5 indirectly regulates RNA methylation and is synthetically lethal with MTAP deletion, a common alteration in drug-tolerance cells with pancreatic, lung, and bladder cancers 135, 136. MRTX1719 137, AMG 193 136, GSK3368715 138, BMS-986504 139 and AMG 193 140 are classical MTA-Cooperative PRMT5 inhibitors, which exhibit strong synthetic lethality in both preclinical models and patients with MTAP-deficient cancers. Notably, flavokawain A, a natural product, has been reported to be a potential PRMT5 inhibitor and is applicable for the treatment of bladder cancer 141. Among all the reported PRMT5 inhibitors, some small molecules, including GSK-3326595 142, 143, JNJ-64619178 144, PRT543 145, PRT811 146, PF-06939999 147, 148 and so on, are currently being assessed in clinical trials** (Table 4)**.

Chemical modification of RNA plays a pivotal role in the development of oligonucleotide-based drugs. Oligonucleotides are characterized by high target affinity, metabolic stability and favorable pharmacokinetic/pharmacodynamic properties. Recent breakthroughs in lipid formulation and GalNAc conjugation of modified oligonucleotides have paved the way for efficient gene delivery and robust, long-lasting gene silencing 149. Fortunately, in other diseases, the action modes of these oligonucleotides include antisense oligonucleotides (ASOs), splice-switching oligonucleotides (SSOs), RNA interference (RNAi) and protein-targeting RNA aptamers 150. Intriguingly, these epigenetic changes can be modulated or reversed not only through chemical compounds but also via non-drug methods. Host system components, including microbiota, circadian clock, metabolic dysregulation and nervous system, significantly influence tumor progression and therapy response 151.

**Figure 4:**
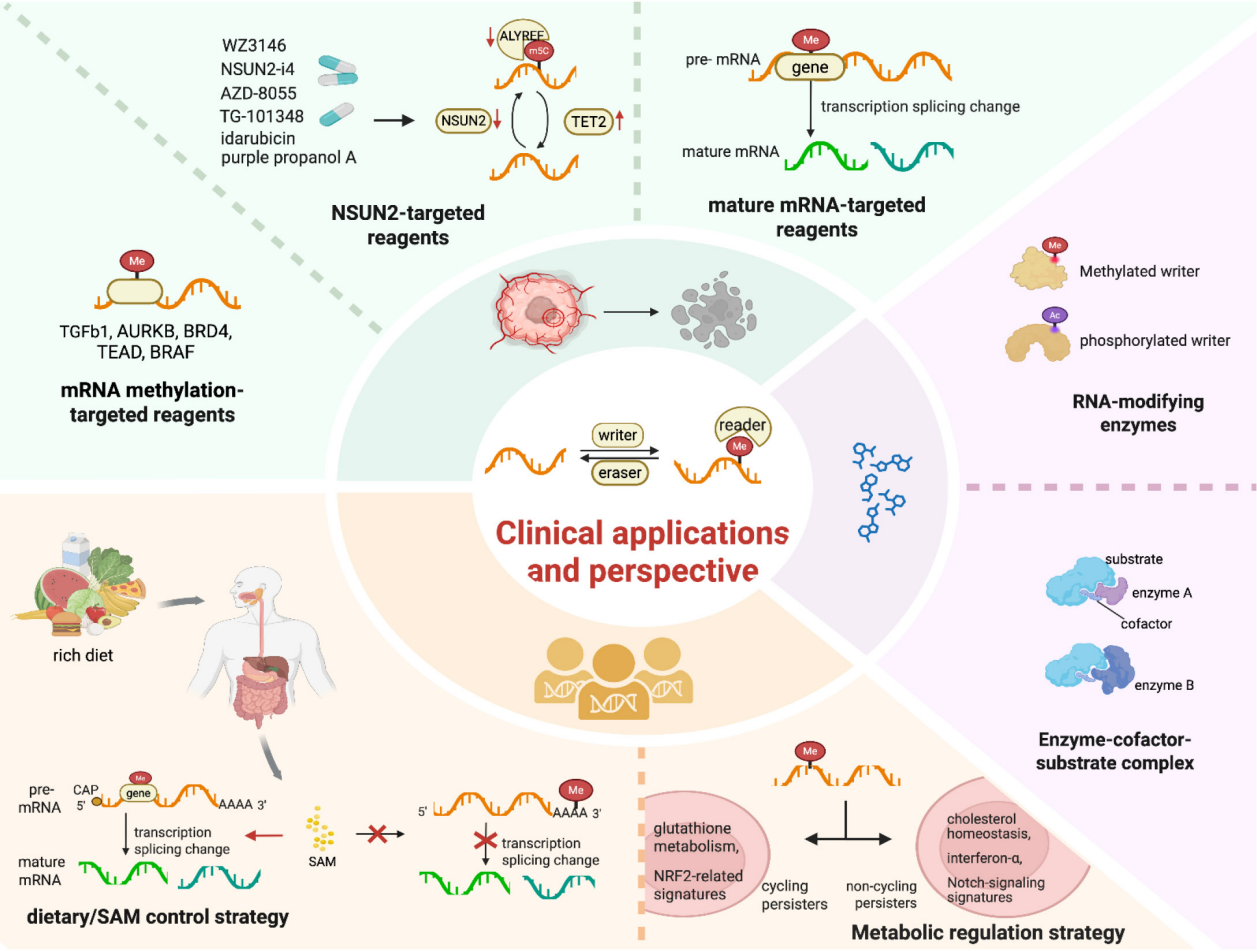
**The Clinical Applications and Perspectives of RNA Methylation. Green:** Reagents targeting mRNA methylation, such as TGFb1 and AURKB; NSUN2-targeted reagents like WZ3146 and NSUN2-siRNA; mature mRNA-targeted reagents. **Yellow:** Dietary/SAM control strategy in the regulation of precursor mRNA splicing, and the generation of diverse mature mRNAs. **Purple:** Modifications of the enzymes or enzyme-cofactor-substrate complexes engaging in RNA methylations. Image made with BioRender.com.

The development of targeted therapeutics against regulators associated with m5C modification, such as activators or inhibitors of methyltransferases and demethylases, is of crucial importance 56 **(Figure 4)**. For instance, compounds such as purple propanol A, idarubicin, WZ3146, AZD-8055 and TG-101348 have been shown to downregulate the expression of NSUN2 and ALYREF while upregulating the expression of TET2 56. The combination therapy involving NSUN2-i4 and PD-1 blockade exhibits a more potent inhibitory effect compared to PD-1 monotherapy 56. RNA methylation on TGFβ1 is preferentially regulated in DTPs. This pre-regulation is especially prominent in cells with inadequate exposure to TKIs, rendering TGFβ1 a viable therapeutic target. Resistance to BRAF inhibitors in melanoma may arise from abnormal changes induced by RNA methylation that affect BRAF mRNA splicing. This results in the production of a truncated BRAF protein that does not respond effectively to small-molecule BRAF inhibition.

Compared with first- and second-generation EGFR-TKIs, the third-generation EGFR-TKI Osimertinib exhibits broad inhibitory effects against EGFR mutations. However, the development of Osimertinib tolerance in DTPs is frequently associated with diverse RNA methylation patterns 152 and enforced cellular heterogeneity 153**.** Utilizing CRISPR technology, AURKB, BRD4 and TEAD have been identified as direct targets epigenetically regulated by RNA methylation **(Figure 4)**. Research has demonstrated that Osimertinib induces a significant time-dependent increase in ROS, which may regulate the proliferative capacity of cycling persister cells 73. This finding supports the hypothesis that cycling persister cells display higher expression levels of glutathione metabolism-related genes and NRF2-related signatures compared to their non-cycling counterparts 73 **(Figure 4)**. In contrast, non-cycling persister cells show elevated expression of genes related to cholesterol homeostasis, interferon-α and Notch signaling signatures. These differences may be linked to various alterations in RNA methylation. For instance, RSL3, an inducer of ferroptosis, undergoes abnormal RNA methylation. This abnormal methylation inhibits GPX4, thereby affecting cycling DTPs, while having no effect on non-cycling DTPs. These findings indicate that these modulators have the potential to be targets for the epigenetically reversing this state.

### 4.3 Non-pharmacological approaches

Non-pharmacological approaches encompass diet, exercise, psychological factors, lifestyle choices and external environments, playing a vital role in tumor control 154. The epigenetic diet (epi-diet) represents a group of dietary bioactives, polyphenols, or other secondary metabolites, mainly present in fruits, vegetables, spices, and other parts of the plants. These dietary bioactives function as promising epigenetic modifiers by targeting the epigenetic modulatory enzymes, or the supply of methyl donors 155. The m6A methylation at a 3ʹ splice site hinders its recognition by splicing factors, thereby inhibiting pre-mRNA splicing in response to a nutrient-rich diet. This mechanism is crucial for regulating gene expression homeostasis, as it controls the production of SAM 156. Methionine is converted into SAM by methyltransferases. SAM plays a crucial role in histone methylation, as well as in the m5C and m6A methylation processes **(Figure 4)**. SAM derived from methionine metabolism promotes the m6A methylation and translation of immune checkpoint proteins, including PD-L1 and V-domain Ig suppressor of T cell activation (VISTA) in tumor cells, contributing to the elevated expression level of these proteins. In two patient-derived xenograft (PDX) mouse models of colorectal cancer (CRC) driven by RAS mutations (KRASG12A or NRASQ61K), dietary methionine restriction alone induced significant tumor regression and potentiated the efficacy of 5-FU treatment 157. Nutritional status and intracellular signals coordinate at the epigenetic level to modulate gene expression via metabolite pool adjustments, enabling cancer cells to rapidly adapt to the changing environmental conditions. For example, metabolites can function as methyl donors for methylation modifications. In a metabolite-limited diet, increased SAM, as a methyl donor, can act as a signal molecule detected by the SAM sensor upstream of mTORC1 (SAMTOR), thus alleviating inhibition of the mTOR pathway due to environmental stressors. In addition, environmental stress can epigenetically modulate the β-adrenergic receptors (β-ARs)/CCL2 axis, thereby inducing anti-tumor immunity and enhancing the sensitivity of immunotherapy against liver cancer in murine models. In contrast, exercise-induced activation of the IL-15/IL-15Rα axis promotes anti-tumor immunity in pancreatic cancer, potentially through mechanisms involving RNA methylation. The dietary methionine restriction or inhibition of the m6A-specific binding protein YTHDF1 could inhibit tumor growth by restoring CD8+ T cell infiltration. This strategy also synergizes with PD-1 blockade, enhancing tumor control *in vitro* and *in vivo* across multiple mouse models 100.

## 5. Conclusion and perspectives

In some sense, cancer can be conceptualized as an emerging entity that exploits the host ecological dysregulation, analogous to embryogenesis, aiming to establish new homeostasis 158. Consequently, tumors should be considered neo-organs with distinct functional and metabolic patterns. Moreover, the tumor environment extends beyond the tumor itself and becomes an integral part of the host organism. External treatments like TKIs significantly disrupt this balance, triggering stress responses in tumor cells. In turn, tumor cells extensively rely on epigenetic modifications for their adaptation and survival. Vice versa, the dynamic and reversible nature of epigenetic alterations that define novel cell states, also provides a promising opportunity for reversing drug tolerance and restoring drug sensitivity.

It is urgent and possible to address the three issues raised previously, albeit with great effort. For identifying the central nodes of RNA methylation modifications triggered by the ISR upon drug treatment and their contributions to ISR proteomics generation, future research should employ multi-omics approaches that integrate RNA methylation profiling, transcriptomics and proteomics during drug-induced ISR 48, 159. Priority should be given to investigating key RNA methylation enzymes (e.g., METTL3, FTO) and their target mRNAs within critical signaling hubs such as the PERK-eIF2α pathway. The dynamic mapping of RNA methylation changes to downstream protein expression patterns, coupled with experiments using specific methyltransferase/demethylase inhibitors in combination with drug treatment (to monitor ISR proteomic outputs), or combination with epi-diets 155, will facilitate the precise pinpointing of these central regulatory nodes. For elucidating how methylation modifications on tRNA and mRNA drive the biogenesis of RNA-derived fragments (e.g., tiRNAs, tRFs), advanced single-molecule imaging techniques should be combined with high-throughput sequencing of specialized RNA fragments (e.g., targeted tRF/tiRNA sequencing) to dissect the spatiotemporal dynamics of tRNA/mRNA methylation during fragment generation. Critical lines of inquiry include exploring the role of stress-activated enzymes (e.g., angiogenin in tRNA cleavage) and how site-specific methylation primes tRNA/mRNA for cleavage (e.g., whether methylation at particular residues enhances tRNA susceptibility to angiogenin-mediated fragmentation), while CRISPR-mediated mutagenesis of key methylation sites on tRNA/mRNA will further clarify the causal link between these modifications and fragment biogenesis. For determining the technologies/models enabling visualization of dynamic RNA epigenome profiles, as well as the optimal barcoding tracers and targets, live-cell imaging systems, which integrate fluorescently tagged RNA methylation enzymes (e.g., GFP-METTL3) with RNA fluorescence *in situ* hybridization for methylated RNA motifs, offer a direct means to visualize real-time epigenome dynamics. For lineage tracing, multiplexed cellular barcoding systems incorporating RNA methylation-sensitive tags (where barcode readouts correlate with changes in methylation status) can effectively track clonal evolution, with ideal tracing targets including highly dynamic methylation sites on stress-responsive mRNAs (e.g., ATF4 mRNA) and conserved tRNA methylation sites critical for tiRNA/tRF production. Additionally, patient-derived organoid models (with matched genetic backgrounds) coupled with single-cell RNA methylation sequencing represent powerful platforms for monitoring RNA epigenome plasticity in physiologically relevant contexts.

The genes involved in drug tolerance are diverse, complex and multifaceted. Therefore, it is imperative to elucidate the state of primed-state cells before and after treatment using appropriate models. Time-series sampling to characterize epigenomic trajectories, as well as monitoring tumor evolution through single-cell epigenomics in circulating tumor cells (CTCs) of patients via LC-ESI-MS/MS analysis of RNA methylation, are valuable approaches. Furthermore, multi-integrated real-time transcriptional reporters allow us to analyze the modulated global transcription rate over time, taking into account both dynamic and static heterogeneity. These analyses are consistent with the ATP metabolism-related cell-cycle markers derived from static heterogeneity, which can assist in validating the DTP state. For lineage tracing, cellular barcoding techniques and imaging-based approaches, along with patient-derived xenograft models or *in vivo* organoid models, can be integrated with bulk RNA-seq data from public datasets. These integrated methods can be used to construct predictive models, with the mapping of RNA methylation modifications serving as a powerful and robust strategy that has been widely applied to *in vitro* and *in vivo* model systems 160-170** (Table 3)**. Additionally, high-throughput technologies, in conjunction with CRISPR-CAS9, will contribute to identifying specific epigenetic vulnerabilities that could be exploited as potential therapeutic targets.

Notably, epigenetic modifications are commonly shared in macromolecules like DNA, RNA and histones, including methylation, acetylation, oxidation and so on. Clinically, DNA/histone modification-targeted therapies have been broadly applied in pre/early clinical trials. The diverse and extensive functions of RNAs in gene regulation provide opportunities for the computational design of targeted interventions. Among these epigenetic modifications, methylation, a well-known and important one, has attracted significant attention. Consequently, the involved methyltransferases and demethylases, such as METTL3, METTL4, FTO, ALKBH5 and YTHDF2, are emerging as viable targets included in clinical trials, as shown in **Table 4** 123, offering promising prospects in conquer of drug tolerance tumor cells.

However, the redundancy and compensatory mechanisms inherent in biological systems introduce a layer of complexity to epigenetic regulation. Multiple enzymes may catalyze the same modification on a specific substrate or act on different substrates, leading to comparable functional outcomes. For example, the combined application of FTO inhibition and anti-PD-1 blockade can mitigate immunotherapy resistance in melanoma patients undergoing TKI-targeted therapy or immunotherapy by targeting RNA epigenetics. The precise identification and characterization of these modifications hold great promise for the development of novel diagnostic tools and targeted therapies. Tailoring treatment strategies to an individual unique and dynamic RNA modification profile is crucial for predicting treatment responses, preventing treatment failure, and adapting therapies in a timely manner. Nevertheless, a significant challenge lies in implementing a team-based medicine approach. Just as understanding drug-tolerant tumor cells is complex, leveraging population-level behavior to develop innovative therapeutic strategies remains a formidable obstacle.

## Figures and Tables

**Table 1:** Characteristics of reviewed RNA modifications

RNA methylation	Writer	Reader	Eraser	Ref.
m1A	TRMT6/61A/61B, TRMT10B/10C, NML	YTHDF1-3, YTHDC1	FTO, ALKBH1/3/7	171
m3C	METTL2A/B, METTL6, METTL8	-	ALKBH1/3	172-176
m5C	NSUN1-7, DNMT2	ALYREF, YBX1, FMRP,	ALKBH1, TETs	171
m6A	METTL3/5/14/16, WTAP, KIAA1429, RBM15, ZC3H13, VIRMA, HAIKAI, ZCCHC4	YTHDF1-3, YTHDC1-2, IGF2BP1-3, hnRNPA2B1, eIF3, PRRC2A	FTO, ALKBH5	177
m7G	RNMT, METTL1/WDR4, WBSCR22/TRM112	eIF4E, CBC	TGS1, H29K9me3	171
Ψ	DKC1, PUS1/7, TRUB1/2, RPUSD3/4	-	-	171

**Table 2:** RNA Methylation Alterations under Drug Stress Mediate Tumor Cells Tolerance

RNA methylation	Regulator	Drug/tumor	Effects	Refs.
**DTPs**				
m5C	NSUN2 (writer)	Geftinib/NSCLC	Enhance OSOX1 translation;	152
	NSUN2 (writer) ALYREF (reader)	Sorafenib/HCC	Maintain SLC7A11 stability	62
m6A	METTL3 (writer)	Anlotinib/OSCC	Increase FGFR3 expression; Inactivate the FGFR3/AKT/mTOR signaling pathway.	178
	FTO (eraser)	Nilotinib/leukemia	Enhance mRNA stability of proliferation/survival transcripts bearing m6A	65
**immune cells**				
m6A	FTO (eraser)	PD-L1/melanoma	Alter m6A methylation in the critical protumorigenic melanoma cell-intrinsic genes including PD-1, CXCR4 and SOX10	64
	FTO (eraser)	PD-L1/colon cancer	Incerase PD-L1 expression	89
	FTO (eraser)	HMA/AML	Alter LILRB4 expression to modulate T cell cytotoxicity	90
	METTL3 (writer)	PD-L1/Bladder Cancer	Resist the cytotoxicity of CD8+ T cells	97
	METTL3 (writer)	PD-L1/OSCC	Activate CD8+ T cells	179
	IGF2BP1 (reader)	PD-L1/colon cancer	Reduce CD8 + T cells-mediated tumor cytotoxicity	98
	ALKBH5 (eraser)	PD-L1/Intrahepatic cholangiocarcinoma	Promote the expression of PD-L1 on monocytes/macrophages and decrease the infiltration of myeloid-derived suppressor-like cells	180

**Table 3:** Current Technologies for Detecting/mapping RNA Methylation

Technology	Targets	Technology Principle	Limitations	Refs
**Global RNA Methylation Detection Methods**				
LC-MS/MS	m6A, m7G, m1A, m5C, Nm	Nucleotide separation; MS ionization/quantification	Costly equipment; complex data analysis	160, 161
Dot Blot	m6A, m1A, m5C, m7G	Membrane-immobilized RNA; antibody binding	Semi-quantitative; no site resolution	162
2D-TLC	m6A, m5C, m1A, Nm	Two-dimensional separation; ³²P labeling and autoradiography	Radioactive reagent dependency; semi-quantitative	163, 164
RiboMethseq	Nm	Nm detection via alkaline resistance and fragment sequencing	<50nt RNA failure; hydrolysis risk	6, 167
MeRIP-seq	m6A, m1A, m5C	Methylated RNAimmunoprecipitation; fragmentation and enriched sequencing	Lower resolution; antibody variability	168, 169
**Transcriptome-wide mapping analysis methods**				
miCLIP	m6A, m6Am	UV crosslinking; methylated fragment immunoprecipitation	Complex workflow	159, 168
iCLIP	m5C, m6A	UV crosslinking; followed by immunoprecipitation; sequencing analysis	Enzymes/Modification limits; complex workflow	181
SMRT	m6A, m5C, m1A, m7G, Nm	Single-molecule RNA sequencing by polymerase kinetics	High cost; complex data analysis	161, 182
Bisulfite RNA sequencing	m5C	Bisulfite C-to-U conversion; sequence comparison; m5C mapping	m5C restriction; high RNA input	165, 166, 181
Bisulfite-free mapping	m5C	Single-base m5C detection; hm5C oxidation; TET conversion	Multiple oxidation steps involved	181, 183
Aza-IP	m5C, m6A	Azacytidine incorporation; immunoprecipitation-based enrichment; high-throughput sequencing	Enzymes/Modification limits; antibody cross-reactivity	181
AlkAnilineSeq	m7G, m3C	Alkaline hydrolysis; 5'-primer ligation enrichment	Complex workflow	181
DART-Seq	m6A	APOBEC1-YTH edits; mutation sequencing	APOBEC1 off-target risks	161, 184
ONT sequencing	m1A, m7G, Nm, m5C, m6A	Nanopore RNA sequencing; translocation-induced current modulation	High sequencing error rate; low throughput; high RNA input	164
RiboMethSeq	Nm	5'-ribose bond protection; detection via protection analysis	Nm detection limits	181
RBS-Seq	Pseudouridine (Ψ)	Bisulfite-induced adduct rearrangement; base skipping	Specific reaction condition limits	181
CMCT-based methods	Ψ	CMCT reaction; reverse transcription (RT) stops/mutational signatures	Moderate Ψ site overlap across studies	181
m7G-MaP-seq	m7G	NaBH₄ reduction; RT mutation sequencing	Chemical treatment affects RNA structure	181
NOSeq	m6A	Nitrous acid resistance; targeted quantification	Chemical treatment affects RNA integrity	181
**Site-specific methylation detection approaches**				
SELECT	m1A, Nm, m6A	Extension/ligation-based capture and qPCR	Cis-elements dependency; low ribosome efficiency	185
MAZTER-seq	m6A	ACA motif sequencing for MazF-resistant level detection	ACA motif restriction; m6Am incompatibility	161, 186
SCARLET	m6A, m5C, m1A	Site-specific cleavage; radioactive labeling; TLC separation	Radioactive reagent dependency; complex workflow; no *de novo* quantification	164, 187
HRM	m6A, m5C	Methylation thermal shift quantification via HRM	High-quality RNA requirement; pre-validated locus dependency	188
RT-qPCR-based assay	m6A, m1A	Detect site-specific methylation via qPCR of RT stops	Targeted site restriction	189
SELECT, LEAD-m6A-seq	m6A	m6A discrimination by Bst DNA polymerase during RT	Low-throughput (SELECT) or locus-specific (LEAD-m6A-seq) limits	181
m6A-SEAL	m6A	FTO oxidation; DDT-thiol biotinylation	Multiple reaction steps involved	181
ARM-Seq, DM-tRNA-Seq	m1A, m3C, m1G	RNA modification detection: native vs. AlkB templates	AlkB-group enzymes limits	181
Pseudo-seq	Ψ	CMC-induced RT termination; adduct sequencing	Novel transcript discovery limits; CMC restriction	52, 190
TRAC-Seq, BoRed-seq	m7G	NaBH₄ reduction via cleavage and RT-stop mapping (TRAC-Seq) or biotinylation and affinity enrichment (BoRed-seq)	Complex procedures (TRAC-Seq) context debate (BoRed-seq, for miRNA)	181
**Hybrid transcriptome-wide/site-specific analysis methods**				
SAM analogs	m6A	Clickable SAM incorporation + biotin enrichment for sequencing	Low transfer efficiency; complex workflow	181
Engineered RT enzymes	2'-O-Me RNA residues, m6A	RNA modification detection via engineered RT activity	Some modifications uncoupled from NGS	181
Mg^2+^ modulation	m1A, m1G, m3C	Mg²⁺ regulation enhances RT arrest/misincorporation	Precise reaction condition optimization	181
4Se-dTTP	m6A	4Se-dTTP induces m6A-specific RT termination	Synthetic dNTP artifact risk	181
2OMe-seq	Ribose 2'-O-Me	AMV RT arrest under low dNTP	Specific RT/dNTP condition limits	181
m6Am-Exo-Seq	m6Am	5'-capped RNA enrichment + anti-m6A immunoprecipitation	Complex multi-step procedure	181

**Table 4:** Clinical Trials of Targeting RNA Methylation in Tumor (update in 30^th^ August 2025)

Drug		Study type	Condition/disease	Primary outcome	Phase	Statue	Study results	NCT
**RNA methyltransferase inhibitor**								
METTL3 Inhibitor	STC-15	Interventional	Advanced Malignant Tumor Resistant to Standard of Care Treatment	AEs	I	Completed	No results posted	05584111
METTL3 Inhibitor	STC-15	Interventional	In combination with toripalimab in locally advanced and unresectable or metastatic NSCLC, melanoma, endometrial cancers and HNSCC	Safety and tolerability of STC-15 in combination with toripalimab	I/II	Recruiting	-	06975293
METTL3 Peptide Inhibitor	-	Observational	Bladder Cancer Prostate CancerKidney Cancer	METTL3 and ANGPTL2 Expression Levels	-	Not yet recruiting	-	06762925
MAT2A Inhibitor	S095035	Interventional	Advanced/Metastatic Solid Tumors with Homozygous Deletion of MTAP	DLTs, AEs, SAEs	I	Recruiting	-	06188702
MAT2A Inhibitor	IDE397	Interventional	Advanced Solid Tumors Harboring MTAP Deletion	DLTs, MTD	I	Recruiting	-	04794699
MAT2A Inhibitor	SYH2039	Interventional	Advanced Solid Tumors	DLTs	I	Recruiting	-	06568614
MAT2A Inhibitor	ISM3412	Interventional	Advanced /Metastatic Solid Tumors with Confirmed Homozygous MTAP Deletion	DLT, AEs, RP2D	I	Recruiting	-	06414460
MAT2A Inhibitor	AG-270	Interventional	Advanced Solid Tumors or Lymphoma with MTAP Loss	DLTs	I	Terminated	-	03435250
PRMT5 Inhibitor	GSK- 3326595	Interventional	Early Stage Breast Cancer	CCCA (2 years)	II	Completed	No results posted	04676516
PRMT5 Inhibitor	AZD3470	Interventional	Advanced/Metastatic Solid Tumors with MTAP Deficient	AEs, SAEs, DLT	I/II	Recruiting	-	06130553
PRMT5 Inhibitor	JNJ-64619178	Interventional	B Cell Non-Hodgkin Lymphoma (NHL), Solid Tumors	DLTs	I	Active, not recruiting	-	03573310
PRMT5 Inhibitor	PRT811	Interventional	Advanced Solid Tumors, CNS Lymphoma, and Recurrent High-Grade Gliomas	DLT, MTD	I	Completed	Doubled apoptosis rate in PRMT inhibitor group vs control; first observation of cross-talk between different arginine methylation types in PRMT inhibitor-treated clinical samples 191	04089449
PRMT5 Inhibitor	AZD3470	Interventional	Relapsed/Refractory haematologic Malignancies	AEs, SAEs, DLTs	I/II	Recruiting	-	06137144
PRMT5 Inhibitor	BGB-58067	Interventional	Advanced Solid Tumors	AEs, SAEs, MTD, MAD, ORR	I	Recruiting	-	06589596
PRMT5 Inhibitor	PRT543	Interventional	Advanced Solid Tumors and Hematologic Malignancies	DLT, MTD	I	Completed	56 R/M ACC patients got PRT543 (35/45 mg, n=28 each); 23% had grade 3 related AEs; mPFS=5.9m; CBR=57%, ORR=2%, 70% stable disease; PRT543 was tolerable, with limited observed efficacy in R/M ACC 145	03886831
PRMT5 Inhibitor	BMS-986504	Interventional	Recurrent Glioblastoma	DLTs, TEAEs	Early I	Recruiting	-	06883747
PRMT5 Inhibitor	BMS-986504	Interventional	Advanced Solid Tumors with Homozygous MTAP Deletion	Cmax, Tmax	I	Recruiting	-	06672523
PRMT5 Inhibitor	PF-06939999	Interventional	Advanced/Metastatic NSCLC, Urothelial Carcinoma, HNSCC	DLT, TEAE	I	Terminated	6 mg daily (RDE) hits PD target (78% plasma SDMA reduction) and balances efficacy with low ≥grade 3 thrombocytopenia risk 192	03854227
PRMT5 Inhibitor	PEP08	Interventional	advanced or metastatic solid tumors harboring MTAP deletion	AE, SAE, DLT	I	Not yet recruiting	-	06973863
PRMT5 Inhibitor	AMG 193	Interventional	Advanced NSCLC	OR, TEAEs	II	Recruiting	-	06593522
PRMT5 Inhibitor	AMG 193	Interventional	Advanced MTAP-null Solid Tumors	DLT, TEAE, ORR	I/II	Recruiting	-	05094336
PRMT5 Inhibitor	S095035	Interventional	Advanced or Metastatic Solid Tumors with Deletion of the MTAP Gene	DLTs, AEs, SAEs	I	Recruiting	-	06188702
PRMT5 Inhibitor plus MAT2A Inhibitor	AMG 193 plus IDE397	Interventional	Advanced MTAP-null Solid Tumors	DLTs, TEAEs, SAEs, OR	I/II	Active, not recruiting	-	05975073
PRMT5 Inhibitor	AMG 193	Interventional	Advanced Gastrointestinal, Biliary Tract, or Pancreatic Cancers with Homozygous MTAP-Deletion	DLT, TEAE, SAE	I	Recruiting	-	06360354
PRMT5 Inhibitor	AMG 193	Interventional	Advanced Thoracic Tumors with Homozygous MTAP-deletion	DLT, TEAE, SAE	I	Recruiting	-	06333951
PRMT5 Inhibitor	IDE397	Interventional	Advanced Solid Tumors Harboring MTAP Deletion	DLTs, MTD	I	Recruiting	-	04794699
PRMT5 Inhibitor	TNG456	Interventional	Solid Tumors with MTAP Loss	MTD	I/II	Recruiting	-	06810544
PRMT5 Inhibitor	GH56 Capsule	Interventional	MTAP-Deleted Advanced Solid Tumors	MTD	I	Recruiting	-	06796699
PRMT5 Inhibitor	TNG908	Interventional	MTAP-deleted Solid Tumors, Including Glioblastoma	MTD, Efficacy	I/II	Active, not recruiting	-	05275478
PRMT5 Inhibitor	TNG462	Interventional	Advanced/Metastatic Solid Tumors with MTAP deletion	MTD, DS	I	Recruiting	-	05732831
PRMT5 Inhibitor	TNG462 plus RMC-6236/ RMC-9805	Interventional	MTAP loss and RAS mutant metastatic PDAC, locally advanced/metastatic NSCLC	MTD	I, II	Recruiting	-	06922591
PRMT5/MTA inhibitor	MRTX1719	Interventional	Advanced Solid Tumors with Homozygous MTAP Deletion	ORR, DOR	I/II	Recruiting	-	05245500
PRMT5 Inhibitor	BGB-58067	Interventional	Advanced Solid Tumors with MTAP Deficiency	AE, SAE, MTD, MAD	I	Recruiting	-	06589596
PRMT5 Inhibitor	BAY3713372	Interventional	MTAP-deleted Solid Tumors	TEAEs, TESAEs, DLTs	I	Recruiting	-	06914128
PRMT5 Inhibitor	AZD3470	Interventional	Relapsed/Refractory Haematologic Malignancies	AEs, SAEs, DLTs	I/II	Recruiting	-	06137144
PRMT1 Inhibitor	CTS2190	Interventional	Advanced/Metastatic Solid Tumors	MTD, ORR	I/II	Recruiting	-	06224387
**Oligonucleotides directly targeting RNA**								
ASO Inhibitor	IONIS-STAT3Rx	Interventional	Advanced lymphoma	MTD, Safety	I/II	Completed	AZD9150 is safe at 3 mg/kg (common AEs: transaminitis, fatigue, thrombocytopenia); 3 mg/kg is recommended Phase 2 dose, and AZD9150 shows efficacy in pretreated DLBCL 193	01563302
ASO Inhibitor	EZN-2968	Interventional	Advanced Solid Tumors With Liver Metastases	Determine the Modulation of HIF-1α mRNA in Pre- and Post-EZN-2968 Tumor Biopsies	I	Completed	-	01120288
ASO Inhibitor	EZN-2968	Interventional	Advanced Solid Tumors or Lymphoma	MTD	I	Completed	No results posted	00466583
Interfering RNA	CALAA-01	Interventional	Solid Tumors Refractory to Standard-of-Care Therapies	MTD	I	Terminated	Systemic siRNA via targeted nanoparticles in melanoma: Post-treatment biopsies show intracellular nanoparticles with dose-correlated amounts, with reduced RRM2 mRNA/protein vs pre-dose 194	00689065
**Diets**								
SAM	anti-PD-1/ PD-L1 antibodies+ S-adenosyl-methionine	Observational	Advanced-Stage Hepatocellular Carcinoma	AEs, SAEs	-	Recruiting	-	05701553
SAM	Supportive Care	Interventional	Colorectal CancerLiver MetastasesLiver Metastasis Colon Cancer	Efficacy Preventing Liver Injury	II	Not yet recruiting	-	06258525
Methionine-restricted diet	Dietary Supplement	Interventional	Lung Cancer, Prostate Cancer, Breast Cancer	Safety of Combined methionine-restricted diet plus RT	-	Terminated	-	03574194
Methionine-restricted diet	Dietary Methionine Restriction	Interventional	Recurrent and/or Progressive Glioblastoma	Time to Disease Progression	I	Terminated	-	00508456
Methionine-restricted diet	Dietary Supplement	Interventional	Metastatic Triple Negative Breast Cancer	ORR	II	Terminated	-	03733119

## Abbreviations

ALKBH: AlkB homolog
ALYREF: Aly/REF export factor
ATF: Activating transcription factor
ATM: Ataxia-telangiectasia mutated
ASO: Antisense Oligonucleotide
BAK: BCL2 antagonist/killer 1
BAX: BCL2 Associated X
BCL2: B-cell lymphoma 2
BCL2L1: BCL2 Like 1
BIM: BCL2 Interacting Mediator of cell death
BRCA1: Breast Cancer gene 1
CDS: Coding sequence
CMTR1: Cap methyltransferase 1
CRC: colorectal cancer
CS1: Bisantrene
CS2: Brequinar
CTCs: circulating tumor cells
CTLA-4: Cytotoxic T-lymphocyte associated protein 4
CTLs: cytotoxic lymphocytes
CXCR4: CXC chemokine receptor type 4
DNMT1: DNA methyltransferase 1
DDR: DNA damage response
DTPs: Drug Tolerant Persister cells
EDAC: Epithelial defense against cancer
eEF: Eukaryotic elongation factor
EV: extracellular vesicle
eIF2α: Eukaryotic translation initiation factor 2 alpha
EGFR: Epidermal growth factor receptor
EMT: Epithelial-mesenchymal transition
ERBB3: Erb-b2 receptor tyrosine kinase 3
ETC: electron transport chain
f5C: 5-formylcytosine
FSCN1: Fascin actin-bundling protein 1
FTO: Fat mass and obesity-associated protein
FTSJ1: FtsJ RNA 2'-O-methyltransferase 1
GC: Gastric cancer
GCLC: Glutamate-cysteine ligase catalytic subunit
GPX4: Glutathione peroxidase 4
HCC: Hepatocellular carcinoma
HIF1α: Hypoxia-inducible factor 1 alpha
HNSCC: Head and neck squamous cell carcinoma
HRI: heme-regulated inhibitor
H3K36me3: Histone H3 lysine 36 trimethylation
ISR: Integrated stress response
IFN: Interferon
IFNγ: Interferon gamma
IGF2BP: Insulin-like growth factor 2 mRNA-binding protein
m1A: N1-methyladenosine
m3A: N3-methyladenosine
m3C: N3-methylcytidine
m5C: 5-methylcytosine
m6A: N6-methyladenosine
m6Am: N6-methyl-2'-O-methyladenosine
m7G: 7-methylguanosine
MALAT1: Metastasis-associated lung adenocarcinoma transcript 1
MCL1: Myeloid cell leukemia 1
METTL: Methyltransferase-like protein
MRD: Minimal residual disease
mRNA: Messenger RNA
ncRNAs: Non-coding RNAs
NGS: next-generation sequencing
Nm: 2'-O-methylation
NRF2: Nuclear factor erythroid 2-related factor 2
NSUN2: NOP2/Sun RNA methyltransferase 2
PD-1: Programmed cell death protein 1
PD-L1: Programmed death-ligand 1
PDX: patient-derived xenograft
R-2HG: R-2-hydroxyglutarate
RAD51: RAD51 recombinase
RAS-G12V: RAS glycine 12 valine mutation
RNAi: RNA interference
ROS: Reactive oxygen species
rRNA: Ribosomal RNA
SAM: S-Adenosylmethionine
SGs: stress granules
siRNA: Small interfering RNA
SLC7A11: Solute carrier family 7 member 11
SOX10: Sex-determining region Y box 10
SSOs: splice-switching oligonucleotides
STIM1: Stromal interaction molecule 1
TET: Ten-eleven translocation
TGF-β: Transforming growth factor beta
TKI: Tyrosine kinase inhibitor
TNF: Tumor necrosis factor
TNM: Tumor-Node-Metastasis
TRDMT1: tRNA aspartic acid methyltransferase 1
tRNA: Transfer RNA
TRMT: tRNA methyltransferase
tRF: tRNA-derived Fragment
tiRNA: tRNA-derived stress-induced RNA
TUBB4A: tubulin β class Iva
UTR: Untranslated region
VISTA: V-domain Ig suppressor of T cell activation
YBX1: Y-Box binding protein 1
YTHDF1/2/3: YTH domain family protein 1/2/3
ZCCHC4: Zinc finger CCHC-type containing 4
5-hmC: 5-hydroxymethylcytosine
